# Adversarial Defense without *Adversarial Defense*: Enhancing Language Model Robustness via Instance-level Principal Component Removal

**DOI:** 10.1162/TACL.a.43

**Published:** 2025-10-29

**Authors:** Yang Wang, Chenghao Xiao, Yizhi Li, Stuart E. Middleton, Noura Al Moubayed, Chenghua Lin

**Affiliations:** The University of Manchester, UK. yang.wang-27@postgrad.manchester.ac.uk; Automated Analytics, UK; Durham University, UK. chenghao.xiao@durham.ac.uk; The University of Manchester, UK. yizhi.li-2@manchester.ac.uk; The University of Southampton, UK. sem03@soton.ac.uk; Durham University, UK. noura.al-moubayed@durham.ac.uk; The University of Manchester, UK. chenghua.lin@manchester.ac.uk

## Abstract

Pre-trained language models (PLMs) have driven substantial progress in natural language processing but remain vulnerable to adversarial attacks, raising concerns about their robustness in real-world applications. Previous studies have sought to mitigate the impact of adversarial attacks by introducing adversarial perturbations into the training process, either implicitly or explicitly. While both strategies enhance robustness, they often incur high computational costs. In this work, we propose a simple yet effective add-on module that enhances the adversarial robustness of PLMs by removing instance-level principal components, without relying on conventional adversarial defenses or perturbing the original training data. Our approach transforms the embedding space to approximate Gaussian properties, thereby reducing its susceptibility to adversarial perturbations while preserving semantic relationships. This transformation aligns embedding distributions in a way that minimizes the impact of adversarial noise on decision boundaries, enhancing robustness without requiring adversarial examples or costly training-time augmentation. Evaluations on eight benchmark datasets show that our approach improves adversarial robustness while maintaining comparable before-attack accuracy to baselines, achieving a balanced trade-off between robustness and generalization.

## 1 Introduction

Pre-trained language models (PLMs) have exhibited remarkable performance across various fields such as computer vision (Dosovitskiy et al., 2021; Touvron et al., 2021; Khan et al., 2022; Wang et al., 2023; Zhu et al., 2024) and natural language processing (NLP) (Devlin et al., 2018; Liu et al., 2019b; Yang et al., 2019; He et al., 2021a, b; Asl et al., 2023). Although they have achieved great success in a number of fields, their vulnerability to adversarial attacks has unveiled a significant challenge to models’ robustness by adding small human-imperceptible perturbations to normal examples (Sun et al., 2020; Li et al., 2020b; He et al., 2021c; Jha and Reddy, 2023).

Existing adversarial defense methods often demand extensive computational resources, or have limited improvements in adversarial robustness. For example, adversarial training-based methods (Madry et al., 2018; Zhu et al., 2020; Li and Qiu, 2021; Wang et al., 2021a) involve generating perturbations through multiple iterations during training, which significantly increases the computational overhead. Similarly, some ensemble-based techniques leverage the statistical properties of the ensemble to provably certify the robustness (Ye et al., 2020a; Zhou et al., 2021b; Moon et al., 2023b; Zeng et al., 2023), leading to additional costs during both training and inference. An alternative line of defense leverages regularization-based methods (Ishida et al., 2020; Wang et al., 2021a; Liu et al., 2022; Yang et al., 2023b), which are more computationally efficient but tend to show limited improvements in robustness against adversarial attacks (Zhu and Rao, 2023). This disparity highlights the need for more efficient adversarial defense approaches that strike a balance between computational efficiency and robustness enhancement.

To address these challenges, we propose Purified Representation (PuRe) to enhance adversarial robustness without introducing adversarial perturbations during training, either implicitly or explicitly.^1^PuRe is implemented as a module that is integrated directly into the PLM’s architecture. The entire model is then trained using a standard fine-tuning process, requiring no special modifications. At its core, this module leverages Principal Component Removal (Arora et al., 2017) to reshape the embedding space. By removing dominant components, it encourages representations to align more closely with Gaussian-like distributions, which reduces the model’s sensitivity to the targeted perturbations that adversaries often exploit. This transformation strengthens robustness without relying on adversarial example generation or resource-intensive training augmentations, providing an efficient and practical solution for improving adversarial resilience in NLP tasks. The evaluation of PuRe is underpinned by benchmarking eight language understanding datasets, spanning across sentiment analysis, subjectivity status classification, paraphrase identification, textual entailment, and commonsense reasoning. PuRe shows superior textual adversarial defense ability to most tasks, while performing on-par with the baselines in terms of before-attack accuracy, indicating a good trade-off between robustness and generalization.

Our contributions can be summarized as follows:

- We introduce PuRe, a novel, parameter-free module for improving adversarial robustness. Its plug-and-play design allows it to be easily integrated into PLMs and optimized with standard fine-tuning, eliminating the need for costly adversarial training.
- We are the first to empirically demonstrate that making the embedding space more geometrically uniform via Principal Component Removal is a highly effective defense mechanism for PLMs.

## 2 Related Work

The concept of model robustness is twofold. General robustness addresses resilience to natural, unintentional variations arising from real-world noise. Adversarial robustness, on the other hand, addresses resilience to malicious, intentional perturbations. These are carefully crafted by an adversary to be imperceptible to humans yet cause the model to fail (Madry et al., 2018; Alayrac et al., 2019; Wang et al., 2020c; Tsai et al., 2021; Wang et al., 2021c). In this work, we focus specifically on enhancing adversarial robustness.

### 2.1 Adversarial Attacks

The field of adversarial attacks was pioneered by Szegedy et al. (2013) in computer vision, where they demonstrated that visually imperceptible distortions could cause models to misclassify images with high confidence. The computational cost of this initial method spurred the development of more efficient gradient-based attacks, including the Fast Gradient Sign Method (Goodfellow et al., 2014b, FGSM) and Projected Gradient Descent (Madry et al., 2018, PGD).

Transferring adversarial attack methods from computer vision to NLP introduces unique challenges due to the discrete nature of textual data as opposed to continuous pixel values. Thus, NLP-focused adversarial attack research has largely focused on crafting semantics-preserving perturbations. For example, back-translation (Iyyer et al., 2018) generates adversarial examples by translating text back and forth between different languages. Wang et al. (2020b) use GANs (Goodfellow et al., 2014a) to create fluent adversarial texts that closely resemble natural language. Additionally, methods have been developed to identify critical words in text and replace them with synonyms or to introduce character-level perturbations such as typos in letters, numbers, or special symbols (Jin et al., 2020; Maheshwary et al., 2021; Li et al., 2018).

These advancements in adversarial attack methods have driven a deeper understanding of NLP models’ vulnerabilities, motivating the development of robust defense strategies to counteract a wide range of adversarial threats.

### 2.2 Adversarial Defenses

Adversarial defenses in NLP aim to enhance the robustness of models against adversarial perturbations. The primary defense strategies can be classified into four categories: adversarial training-based, perturbation control-based, certification-based, and regularization-based methods.

#### Adversarial training-based methods

involve augmenting the training data with adversarial examples, enabling the model to learn in an environment that simulates attacks in the training process, either implicitly or explicitly (Jin et al., 2020; Morris et al., 2020; Si et al., 2020; Wang et al., 2021a; Hauser et al., 2023). Implicit approaches usually generate perturbations dynamically in the embedding space as a part of the training process, which improves the model’s resilience to a range of adversarial scenarios (Wu et al., 2017; Zhu et al., 2020; Dong et al., 2021; Gao et al., 2023; Latorre et al., 2023). Explicit approaches, on the other hand, involves generating adversarial examples in the input space (text data) using adversarial attack methods (Jin et al., 2020; Li et al., 2020c; Tan et al., 2020; Zang et al., 2020), and these pre-generated adversarial examples will be incorporated into the training pipeline. We refer this explicit adversarial training-based approach as Adversarially-augmented (AdvAug) training (see §5.2). Despite its efficacy and interpretability, the adversarial training-based methods are often computationally intensive due to the need for extensive adversarial example generation and fine-tuning.

#### Perturbation control-based methods

aim to detect and correct adversarial inputs by incorporating mechanisms to recognize potential perturbations (Alshemali and Kalita, 2019; Yoo et al., 2022; Shen et al., 2023; Ali et al., 2023) or by altering the perturbation toward cleaner inputs to limit the adversarial space (Sato et al., 2018; Zhang et al., 2020; Zhou et al., 2021a; Bao et al., 2021). Techniques include spell-checking systems for character-level defenses that correct adversarially manipulated inputs before classification (Alshemali and Kalita, 2019) and word-level defenses that substitute input words with synonyms to neutralize adversarial effects (Ye et al., 2020a; Zhou et al., 2021a; Dong et al., 2021). However, synonym-based methods often face limitations in practical scenarios, where the perturbation sets of potential attacks are usually unknown (Li et al., 2021a).

#### Certification-based methods

provide theoretical guarantees by constructing a perturbation-resistant region around the input space (Wang et al., 2019; Dong et al., 2021; Asl et al., 2023; Moon et al., 2023a; Zeng et al., 2023). Although these methods offer strong theoretical assurances, they typically involve impractical constraints in real-world applications. Certification-based methods can require extensive computational resources and long verification times (Zeng et al., 2023), which may not be feasible in applications with limited computational capacity or real-time processing requirements.

#### Regularization-based methods

add regularization terms to the loss function to improve model robustness without relying on adversarial examples generation or pre-defined synonym sets. For example, Wang et al. (2021a) introduced two regularizers to improve out-of-domain robustness evaluated on adversarial NLI (Nie et al., 2020) and SQuAD (Jia and Liang, 2017) datasets. The first regularizer is an implementation of the Information Bottleneck principle (Tishby and Zaslavsky, 2015) specialized for contextual text representations, and the second regularizer is to minimize the mutual information between the input and the representation. Liu et al. (2022) introduced a “flooding” loss (Ishida et al., 2020), which helps models avoid overconfidence in predictions by maintaining the loss at a specific threshold. Their findings suggest that the flooding method shows promise in defending against adversarial attacks. Yang et al. (2023b) modified the traditional label smoothing technique (Guo et al., 2017) to account for adversarial perturbations, thereby enhancing model resilience. These structure-free approaches offer computational advantages over methods that depend on explicitly generated adversarial data or pre-defined perturbation sets.

### 2.3 Isotropic Latent Space

Isotropy in the context of representation learning refers to the uniform distribution of the directions of vectors in the embedding space, implying that no particular direction is overly dominant (Mu and Viswanath, 2018a). The embeddings spread more evenly across all dimensions, resembling a spherical Gaussian-like distribution where all directions are statistically similar.

Mu and Viswanath (2018a) propose a post- processing algorithm that masks out the top principal components of the data, and show that it improves performance for Word2Vec (Mikolov, 2013) and GloVe (Pennington et al., 2014) embeddings on word similarity tasks. Achieving an isotropic latent space has also been explored in prior work (Li et al., 2020a; Huang et al., 2021; Su et al., 2021), arguing that improving isotropy in the embedding space improves model performance. Similarly, Kernel-Whitening (Gao et al., 2022) employs isotropic transformations to mitigate dataset bias, demonstrating the benefits of a uniform representation space for generalization. More recent approaches such as I-STAR (Rudman and Eickhoff, 2024), which is a differentiable and mini-batch-stable isotropy-based regularization scheme, studies the relationship between fine-tuned model performance and isotropy. Contrary to previous works in NLP, Rudman and Eickhoff (2024) find that further decreasing isotropy improves downstream model performance. While these methods enhance the quality of embeddings for downstream tasks, they often serve as a post-processing step and do not explicitly address adversarial robustness.

On the other hand, PuRe builds on the idea of isotropic representations but shifts the focus towards adversarial robustness. We hypothesize that isotropic transformation can reduce the sensitivity to adversarial perturbations and regularize decision boundaries, providing a more robust defense mechanism. To sum up, we derive several keys to distinguish PuRe from existing adversarial defense methods. (i) PuRe obviates the need for generating adversarial examples, whether implicitly or explicitly, resulting in significant computational savings. (ii) It addresses adversarial vulnerabilities via Principal Component Removal, thereby providing a robust defense mechanism that does not rely on particular attack constraints. (iii) It is a simple, add-on module that can be seamlessly integrated with off-the-shelf PLMs, offering a model-agnostic solution.

## 3 Purified Representation (PuRe)

We propose PuRe (Purified Representation), a method designed to improve adversarial robustness by encouraging isotropy in the representation space (i.e., making embeddings more uniformly distributed across dimensions). This isotropic structure reduces sensitivity to adversarial perturbations and strengthens the stability of decision boundaries. PuRe achieves this through a simple yet effective adaptation of Principal Component Analysis (PCA; Abdi and Williams, 2010) to standardize the latent space. In this section, we detail the design and intuition behind PuRe.

### 3.1 Instance-level Principal Components Removal

The core idea behind PuRe is to reduce the dominance of certain directions in the representation space by removing principal components that capture most of the variance. Traditional PCA typically discards the weakest directions (i.e., principal components with the least variance) to minimize information loss. For example, BERT-whitening (Su et al., 2021) applies PCA to BERT embeddings by discarding less informative dimensions, thereby retaining important textual features and improving performance in semantic similarity tasks. In contrast, PuRe applies PCA in a novel manner, aiming for significant information reduction to enhance adversarial robustness. PuRe subtracts these dominant components from the final layer token-level representations. This results in a representation space that is closer to an isotropic distribution, where all directions carry roughly equal importance (see Figure 4).

PuRe draws inspiration from techniques like SIF embeddings (Arora et al., 2017), which remove the top-1 principal component from static embeddings to capture variance in rogue dimensions (Timkey and van Schijndel, 2021a), making the representation space more isotropic. However, rather than applying Principal Component Removal (PCR) as a post-processing step to the entire corpus, PuRe performs this operation at the instance level, removing projections onto the top-1 principal component of the subspace spanned by individual tokens within a sentence during fine-tuning. We combined with efficient principal component computation via Singular Value Decomposition (SVD; Golub and Reinsch, 1971), enables end-to-end training while achieving an isotropic latent space, which is shown ultimately improving the model’s resilience to adversarial perturbations. Preliminaries of PCA and SVD can be found in Appendix A.

Suppose having final layer token-level embedding **X** ∈ℝ^*n*×*d*^, with a sequence length *n* and embedding dimension *d*. We perform SVD on **X** and get the right singular matrix **V**. The columns of **V** are the corresponding principal components (since SVD directly computes the eigenvector matrix **V**), which are already sorted by descending eigenvalue. We null away the top-1 principal component:^2^

$$X\leftarrow X-(Xv_{1})v_{1}^{⊤}$$ (1)

Eq. 1 is equivalent to removing rank-1 matrix corresponding to largest singular value from **X**:

$$\begin{array}{ll} X-(Xv_{1})v_{1}^{⊤} & =X-(u_{1}\sigma_{1})v_{1}^{⊤}\\ & =(\overset{k}{\underset{i=1}{\sum }}\sigma_{i}u_{i}v_{i}^{⊤})-\sigma_{1}u_{1}v_{1}^{⊤} \end{array}$$ (2)

This operation essentially removes the component of **X** that is in the direction of the largest singular value, represented by $\sigma_{1}u_{1}v_{1}^{⊤}$. The largest singular value, *σ*_1_, and its corresponding singular vectors, **u**_1_ and **v**_1_, capture the most significant mode of variation (or the principal component) in the tokens embedding matrix **X**. Building upon the findings of Mu and Viswanath (2018b), they observe that by post-processing the word representation by eliminating the common parts, the processed word representations is able to capture stronger linguistic regularities (i.e., the semantic similarity of words is well captured by the similarity of the corresponding vector representations). They posit that PCR makes the representations more *isotropic* with stronger self-normalization properties. We then hypothesize that a uniform distribution of embeddings can lead to more stable decision boundaries, because adversarial attacks often seek to exploit the model by finding inputs that cross these boundaries with minimal changes. A more isotropic space might reduce the number of “weak spots” or vulnerabilities that adversarial inputs can exploit. Therefore, if the dominant principal component corresponding to the largest singular value is thought to represent noise or an unwanted signal, its removal can help in focusing on more subtle underlying structures and consequently yield a more distilled and essence-focused representation of the text.

#### 3.1.1 Randomized SVD

Traditional methods for SVD can be computationally intensive, particularly with the increasing size and complexity of data matrices (Wang et al., 2021b; Song et al., 2021, 2022). Addressing this challenge requires approaches that reduce computation time without compromising accuracy.

To compute principal components, we use randomized SVD (Halko et al., 2011, rSVD) that extracts the column space from unilateral random projections. rSVD utilizes randomization to accelerate the process of finding a low-rank approximation of a matrix. This enables efficient processing of large matrices, significantly reducing computational costs, while also mitigating potential adversarial effects (Bingham and Mannila, 2001; Xie et al., 2017; Taran et al., 2019). Following Halko et al. (2011), we adopt a two-stage framework to approximate a low-rank matrix of a given *m* × *n* matrix **A** using randomized algorithms:

##### Step 1.

Compute an approximate basis **Q** with *l* orthonormal columns for the range of **A**, such that **A** ≈**Q** Q^*^A.

##### Step 2.

Given such a matrix **Q**, which is much smaller than **A**, we use it to compute our desired SVD.

Motivated by the Johnson-Lindenstrauss lemma (Johnson, 1984), we explore the preservation of pairwise distances. This lemma demonstrates that such distances among a set of points in a Euclidean space can be approximately maintained when projected into a lower-dimensional Euclidean space. Utilizing this principle, we employ random sampling on the range of **A**. We use a Gaussian random matrix, denoted as **Ω** ∈ℝ^*d*×*r*^, where *r* is a sampling parameter indicating the number of Gaussian random vectors. The orthonormal basis for these vectors yields the desired basis **Q**. This scheme is formally presented in Halko et al. (2011).

The efficiency of the rSVD algorithm derives from the fact that **B** = Q^*^A is relatively smaller in comparison to **A**, where ^*^ represents the conjugate transpose operation (Turnbull and Aitken, 1932). This efficiency is based on the observation that **A** is approximately equal to $A\approx QQ^{*}A=Q(Ũ\Sigma V^{*})$, allowing us to set U=QŨ to obtain a low-rank approximation, resulting in **A** ≈**U** **Σ** V^*^. It is important to note that the randomness only occurs during the computation of **Q** matrix, while Step 2 in the SVD computation remains deterministic when **Q** is given. Following Halko et al. (2011), we employ the subspace iteration method to implement the randomized range finder for obtaining matrix **Q**.

### 3.2 Sentence-level Representation

After obtaining the *purified* token-level representations from the PCR module, we aggregate them to form a single sentence-level representation. To do this, we employ Parameter-Free Self-Attention (PFSA) from Zhai et al. (2023) before the final mean pooling step. PFSA is ideal for this task as it captures global sentence-level features with linear computational complexity and without introducing any trainable parameters. This parameter-free design improves the final semantic representation while mitigating the risk of overfitting. Our ablation study (§5.6) confirms that this approach is more effective and efficient than using mean pooling alone.

**Figure 1:**
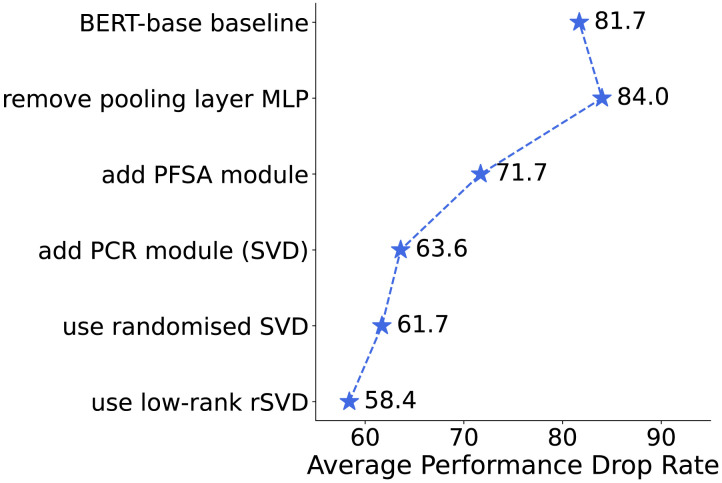
**The development trajectory** of the module design of PuRe. Each line is based on a modification of the immediately preceding line, tested on the SST2 test set.

Finally, Figure 1 shows the development trajectory of the module evolved from a standard BERT-base baseline into a model capable of adversarial defense upon integrating the PuRe module. More details on the ablation experiments supporting this development trajectory can be found in §5.6.

## 4 Experiments

### 4.1 Baselines

We use a diverse set of baselines to benchmark PuRe. For adversarial training-based methods, we include PGD (Madry et al., 2018), FreeLB (Zhu et al., 2020), InfoBERT (Wang et al., 2021a), and TAVAT (Li and Qiu, 2021). For perturbation control-based methods, we adopt DNE (Zhou et al., 2021b) and AdvFooler (Hoang et al., 2024). For certification-based methods, we adopt SAFER (Ye et al., 2020a). For regularization-based methods, we include Flooding-X (Liu et al., 2022) and ALS (Yang et al., 2023b). For consistency and fair comparison, all baselines follow the setup outlined in the TextDefender framework (Li et al., 2021b).

To evaluate the scalability of PuRe, we apply it across a diverse set of model architectures, including encoder-only models such as BERT (Devlin et al., 2018), RoBERTa (Liu et al., 2019b), and DeBERTa (He et al., 2020, 2021a, b); decoder-only models like OPT (Zhang et al., 2022) and Qwen2.5 (Yang et al., 2024; Qwen Team, 2024); and embedding-based models such as BGE (Xiao et al., 2023b) and GIST (Solatorio, 2024). All baselines are fine-tuned using their default settings as described in the original papers. Further details on the baselines are provided in Appendix C, with model architectures, checkpoints, and parameter sizes listed in Appendix D.

### 4.2 Adversarial Attackers for Evaluation

We choose three attackers to evaluate the robustness to adversarial changes. These attackers are leveraged via TextAttack^3^ (Morris et al., 2020) for an extensive comparison between PuRe and the baseline defense strategies. We use default hyperparameters provided by TextAttack library.

#### TextFooler

(Jin et al., 2020) is a black-box adversarial attack method that generates adversarial examples by ranking and replacing important words with semantically and grammatically similar substitutes, aiming to alter model predictions while preserving the fluency of the original text. It demonstrates high attack success rates across NLP tasks like text classification and entailment by using efficient perturbations.

#### TextBugger

(Li et al., 2018) is designed to generate semantic-preserving adversarial texts under both white-box and black-box settings. It uses character- and word-level perturbations to manipulate texts minimally while achieving high attack success rates against real-world NLP applications.

#### PWWS

(Ren et al., 2019) is a black-box adversarial attack approach. It generates adversarial examples by replacing words based on their saliency and classification probability, ensuring minimal semantic and grammatical disruption while significantly affecting model predictions.

While we acknowledge the advancements in attack techniques, TextAttack currently provides limited support for newer methods up to 2021. Therefore, we focused on three well-established, general-purpose attack methods that are widely recognized for evaluating adversarial robustness (Nguyen Minh and Luu, 2022; Wang et al., 2022a, b; Yang et al., 2023a; Zhan et al., 2023; Hu et al., 2023; Yang et al., 2023b; Gao et al., 2023; Shen et al., 2023; Lu et al., 2024; Ji et al., 2024; Zhang et al., 2024; Zhao et al., 2024).

### 4.3 Evaluation Metrics

Following prior studies (Zhan et al., 2023; Zhao et al., 2024), we consider four evaluation metrics to measure the resilience of victim models against the aforementioned adversarial attacks. Considering the diverse evaluation metrics across tasks and varying defensing performance across models, we also adopt performance drop rate (Zhu et al., 2023) to quantify the relative performance decline.

#### Clean Accuracy (Acc)

measures the accuracy of the model on the before-attack dataset. It provides a baseline for how well the model performs without adversarial interference.

#### Accuracy Under Attack (Aua)

evaluates the accuracy of the model when subjected to adversarial examples. A higher Aua indicates better robustness against adversarial attacks.

#### Attack Success Rate (Asr)

is the percentage of adversarial attacks that successfully cause the model to misclassify. A lower Asr signifies a more robust model.

#### Number of Queries (AvgQ)

quantifies the average number of queries made to the model by an adversarial attack to achieve success. A higher number implies the model is harder to attack (Li et al., 2021a).

#### Performance Drop Rate (Pdr)

quantifies the relative performance decline, and provides a normalized measure for comparing different attacks (Zhu et al., 2023). Apdr stands for average Pdr across different attacks.

### 4.4 Datasets

We evaluate PuRe across eight language understanding datasets covering various NLP tasks such as: sentiment analysis, subjectivity status classification, paraphrase identification, textual entailment, and commonsense reasoning. In contrast to other studies (Dong et al., 2021; Bao et al., 2021; Li et al., 2021a; Wang et al., 2022a; Shen et al., 2023; Hu et al., 2023; Zeng et al., 2023; Zhan et al., 2023; Moon et al., 2023b), which often restrict their evaluations to a limited selection of test samples from their datasets, we extend our analysis to include the entire test sets for all eight datasets, ensuring a comprehensive assessment. This broad evaluation approach contrasts with the common practice in the field, where researchers only utilize a small portion of available test data, which may not fully represent the model’s performance across different scenarios.

#### SST2

(Socher et al., 2013) is a sentiment classification dataset of movie reviews.

#### SUBJ

(Pang and Lee, 2004) is a review dataset with sentences labeled as subjective or objective.

#### CR

(Hu and Liu, 2004) is a sentiment classification dataset of customer reviews.

#### MR

(Pang and Lee, 2005) is a dataset containing positive and negative sentences from Rotten Tomatoes movie reviews.

#### MRPC

(Dolan and Brockett, 2005) is a corpus consisting of sentence pairs collected from news-wire articles. Each pair is labeled if it is a paraphrase or not by human annotators.

#### SICK

(Marelli et al., 2014) is a large dataset on compositional meaning, annotated with subject ratings for both relatedness and entailment relation between sentences.

#### SIQA

(Sap et al., 2019) is a commonsense reasoning dataset where the goal is to choose the most appropriate answer from three options to questions about everyday social situations.

#### CSQA

(Talmor et al., 2019) is another multiple- choice question answering dataset that requires different types of commonsense knowledge to predict the correct answers.

Note that the test sets of SIQA and CSQA are not publicly available; we evaluate baselines and PuRe on their validation sets. Table 8 summarizes the statistics of the four single text classification datasets, two text pairs classification datasets, and two multiple-choice classification datasets.

### 4.5 Implementation Details

We take the output vector from the pooling layer and use it to construct a feed-forward neural network. We employ an affine transformation followed by a softmax and cross-entropy for classification. We fine-tune PLMs using AdamW optimizer (Loshchilov and Hutter, 2017) for four epochs.

To keep experiments simple and reproducible, we avoid extensive hyper-parameter tuning and instead apply a light grid search over a small set of commonly used values: batch sizes {8,16,32} and learning rates {1e−5,2e−5,5e−5}. For the three adversarial attackers, all four evaluation metrics are tested on the entire test set for every dataset on sequence classification tasks.

For commonsense reasoning datasets, we follow Branco et al. (2021), converting the multiple-choice task into a sequence-ranking problem, as outlined in Liu et al. (2019a). We process the elements of input pairs separately, generating a score for each, with the maximum score corresponding to the selected answer. More training details can be found in our public source code.^4^

## 5 Results and Analysis

This section compares PuRe to other baselines in several configurations across datasets and attacks. For simplicity, if not specified, we refer the backbone to BERT-base in the following analysis.

### 5.1 Generalization and Robustness

Table 1 presents the experimental results for the BERT-base model with various defense methods. We observe the same general trends across all models, and therefore present the results for BERT-base here and the others in Appendix D.

**Table 1:** Adversarial robustness results with different baselines. **Bold**: the best. Underline: the second best.

**Dataset**	**Method**	**Acc** ↑	**TextFooler**			**TextBugger**			**PWWS**			**Apdr** ↓
			**Aua** ↑	**Asr** ↓	**AvgQ** ↑	**Aua** ↑	**Asr** ↓	**AvgQ** ↑	**Aua** ↑	**Asr** ↓	**AvgQ** ↑	
SST2	Fine-tune	92.09	6.32	93.14	87.07	30.37	67.02	41.19	13.78	85.03	127.57	81.73
	PGD	91.21	12.63	86.15	108.44	36.57	59.90	43.99	22.30	75.56	136.51	73.87
	FreeLB	**92.15**	10.76	88.32	107.30	36.63	60.25	44.32	20.92	77.29	136.10	75.29
	InfoBERT	91.93	8.29	90.98	98.53	31.41	65.83	42.10	19.00	79.33	133.55	78.72
	TAVAT	90.50	12.19	86.53	111.46	36.13	60.07	43.73	24.00	73.48	137.93	73.36
	DNE	86.72	10.94	87.38	105.50	24.11	72.20	46.16	19.67	77.28	104.85	78.97
	SAFER	91.76	7.58	91.74	92.96	32.62	64.45	41.05	14.06	84.68	128.81	80.29
	Flooding-X	91.65	4.12	95.51	81.59	27.62	69.86	39.50	12.63	86.22	127.17	83.86
	ALS	91.32	18.07	80.22	117.18	42.39	53.58	45.42	22.19	75.71	133.40	69.83
	AdvFooler	90.55	11.56	87.23	100.98	39.09	56.83	42.65	17.43	80.75	130.73	74.94
	**PuRe (Ours)**	90.88	**30.37**	**66.59**	**134.01**	**47.17**	**48.10**	**58.52**	**36.02**	**60.36**	**139.97**	**58.35**
SUBJ	Fine-tune	97.40	25.30	74.02	189.74	63.15	35.16	69.19	41.95	56.93	196.06	55.37
	PGD	97.15	46.90	51.72	230.91	77.40	20.33	72.48	60.70	37.52	208.30	36.52
	FreeLB	**97.50**	44.40	54.46	226.76	76.95	21.08	73.76	58.40	40.10	206.93	38.55
	InfoBERT	97.41	35.65	63.40	207.53	72.25	25.82	72.93	51.20	47.43	201.44	45.56
	TAVAT	97.05	50.25	48.22	233.11	78.45	19.17	72.80	62.60	35.50	208.90	34.30
	DNE	95.80	48.45	49.29	223.59	59.45	37.94	84.94	62.40	34.76	136.63	40.74
	SAFER	97.25	32.90	66.17	203.86	68.05	30.03	73.23	46.80	51.88	199.27	49.36
	Flooding-X	97.15	24.20	75.09	189.59	66.35	31.70	72.12	40.40	58.41	195.65	55.07
	ALS	97.45	38.60	60.39	217.69	71.80	26.32	73.80	51.90	46.74	202.45	44.48
	AdvFooler	96.97	35.96	62.92	204.65	70.19	27.62	76.67	49.11	49.36	204.99	46.63
	**PuRe (Ours)**	96.75	**67.85**	**29.87**	**250.60**	**80.05**	**17.26**	**96.61**	**73.80**	**23.72**	**210.42**	**23.62**
CR	Fine-tune	92.28	3.99	95.68	81.16	36.70	60.23	35.10	10.64	88.47	127.97	81.46
	PGD	91.76	14.63	84.06	113.88	54.52	40.58	41.00	21.28	76.81	142.28	67.15
	FreeLB	92.82	11.70	87.39	103.70	53.99	41.83	41.33	17.82	80.80	136.81	70.01
	InfoBERT	**94.15**	10.11	89.27	98.24	48.40	48.59	38.91	15.69	83.33	134.16	73.73
	TAVAT	91.22	15.16	83.38	115.63	57.18	37.32	41.80	25.00	72.59	143.23	64.43
	DNE	88.74	11.81	86.69	116.49	35.16	59.37	**52.12**	18.13	79.50	111.51	75.55
	SAFER	93.09	9.84	89.43	94.43	44.95	51.71	38.23	13.30	85.71	130.17	75.62
	Flooding-X	91.22	3.19	96.50	84.81	44.68	51.02	36.43	10.11	88.92	132.35	78.81
	ALS	91.22	10.11	88.92	99.68	46.81	48.69	39.59	11.44	87.46	128.68	75.02
	AdvFooler	89.64	11.91	86.71	101.37	48.65	45.73	40.33	15.13	83.12	135.76	71.85
	**PuRe (Ours)**	88.82	**37.23**	**58.08**	**138.43**	**57.98**	**34.73**	46.62	**33.51**	**62.28**	**143.87**	**51.69**
MR	Fine-tune	85.64	5.35	93.76	91.59	24.86	70.97	44.25	13.23	84.56	138.90	83.09
	PGD	85.18	11.35	86.67	122.64	36.59	57.05	49.31	22.42	73.68	149.77	72.47
	FreeLB	86.30	7.32	91.52	109.88	30.11	65.11	47.50	17.45	79.78	144.52	78.80
	InfoBERT	**86.59**	8.26	90.47	111.43	32.27	62.73	47.12	18.76	78.33	146.14	77.18
	TAVAT	84.90	11.82	86.08	123.43	34.62	59.23	50.28	23.55	72.27	**151.25**	72.52
	DNE	82.49	7.04	91.46	94.56	14.67	82.22	48.64	15.70	80.86	114.13	84.88
	SAFER	86.30	10.79	87.50	105.78	31.80	63.15	47.23	17.35	79.89	140.78	76.85
	Flooding-X	85.83	3.47	95.96	89.38	26.17	69.51	44.06	11.26	86.89	137.13	84.12
	ALS	85.65	15.38	82.04	116.72	34.99	59.15	50.06	21.20	75.25	142.89	72.15
	AdvFooler	83.28	14.94	82.06	106.91	33.18	60.16	50.19	20.87	74.94	144.76	72.39
	**PuRe (Ours)**	85.64	**25.89**	**69.74**	**135.57**	**40.06**	**53.18**	**58.87**	**31.61**	**63.05**	151.08	**62.03**
MRPC	Fine-tune	84.40	2.32	97.25	124.00	3.25	96.15	72.84	4.41	94.78	250.53	96.06
	PGD	84.06	9.86	88.28	205.38	11.25	86.62	101.98	16.12	80.83	282.70	85.24
	FreeLB	85.45	11.48	86.57	212.41	11.65	86.36	107.19	17.91	79.04	283.42	83.99
	InfoBERT	**85.91**	5.22	93.93	168.33	6.72	92.17	89.11	9.91	88.46	269.48	91.52
	TAVAT	84.29	8.70	89.68	**229.16**	10.43	87.62	106.60	17.22	79.57	**289.42**	85.63
	DNE	73.04	**21.97**	**69.92**	186.46	4.70	93.58	82.30	**19.19**	**74.14**	227.71	79.07
	SAFER	84.46	3.07	96.36	121.40	3.30	96.09	70.37	4.75	94.37	249.26	95.61
	Flooding-X	82.03	5.04	93.85	141.62	5.51	93.29	81.77	8.52	89.61	260.67	92.25
	ALS	83.77	4.06	95.16	149.17	6.03	92.80	82.89	9.10	89.13	260.25	92.36
	AdvFooler	83.46	4.67	94.40	150.98	7.64	90.85	90.13	6.84	91.80	267.37	92.35
	**PuRe (Ours)**	82.20	17.22	79.07	226.10	**16.29**	**80.20**	**107.73**	18.55	77.45	273.21	**78.89**
SICK	Fine-tune	86.93	20.81	76.06	117.47	26.42	69.61	50.19	25.11	71.11	183.30	72.26
	PGD	86.24	37.18	56.89	140.17	33.33	61.36	53.01	**40.28**	53.30	194.52	57.18
	FreeLB	**88.79**	28.05	68.41	125.13	31.80	64.19	52.57	30.98	65.11	188.02	65.90
	InfoBERT	88.73	26.97	69.61	125.24	30.68	65.43	51.62	28.76	67.59	186.43	67.54
	TAVAT	87.85	35.51	59.58	**147.97**	33.80	61.53	53.65	35.55	59.54	191.88	60.21
	DNE	82.13	29.49	63.94	88.77	19.38	76.31	54.71	25.53	68.91	141.88	69.80
	SAFER	86.85	27.78	68.01	135.33	34.10	60.74	52.78	33.57	61.35	193.89	63.37
	Flooding-X	86.53	24.75	71.40	119.77	23.99	72.27	48.19	26.68	69.16	184.09	70.95
	ALS	86.28	29.17	66.19	125.74	27.90	67.66	47.90	28.11	67.42	186.23	67.09
	AdvFooler	85.73	30.91	63.94	140.97	33.79	60.59	53.46	34.49	59.77	194.49	61.43
	**PuRe (Ours)**	84.32	**38.67**	**54.12**	143.56	**38.50**	**54.32**	**56.74**	39.58	**53.04**	**195.34**	**53.85**

PuRe performs on par with the baselines in terms of before-attack accuracy, indicating a good trade-off between robustness and generalization. This trade-off (i.e., higher after-attack accuracy and slightly lower before-attack accuracy) lies in the role of dominant directions in the representation space. High-frequency tokens tend to align with top principal components (Arora et al., 2017). Removing these components (most of which are not useful discriminative features, with only a small fraction lying in the dominant vector space) inevitably leads to a minor decrease in clean accuracy, since some discriminative information is lost. While PuRe may cause a slight drop in accuracy on clean data, it typically yields much more resilient decision boundaries and improved robustness to adversarial perturbations.

We observed a notable variation in the Apdr scores across six datasets when subjected to adversarial attacks. Specifically, datasets such as SST2, MR, and MRPC exhibit higher Apdr values (58.35%, 62.03%, 78.89% respectively), suggesting these are more challenging to defend compared to SUBJ, CR, and SICK, which demonstrated lower Apdr values (23.62%, 51.69%, 53.85% respectively). This variability in resilience may be attributed to inherent dataset characteristics, including the complexity of the text, the diversity of linguistic expressions, and the nature of the tasks involved. For instance, simpler datasets like SST2 might be more susceptible to semantic shifts caused by adversarial perturbations due to their straightforward linguistic structures. Conversely, datasets like SICK, involving more complex semantic relationships, might inherently diffuse such attacks more effectively. Thus, our subsequent analysis will primarily focus on SST2, MR, and MRPC datasets.

### 5.2 Adversarially Augmented Training

We perform an AdvAug experiment on the SST-2 dataset by augmenting the training set with adversarial examples that preserve the original labels. Each training sample is initially paired with one adversarial counterpart, resulting in a 2x dataset. To investigate the effect of larger-scale augmentation, we further expand the dataset by generating up to four distinct adversarial examples per input, creating datasets up to 5x the original size. These augmented datasets are then used to fine-tune a BERT-base model under same training configurations.

**Figure 2:**
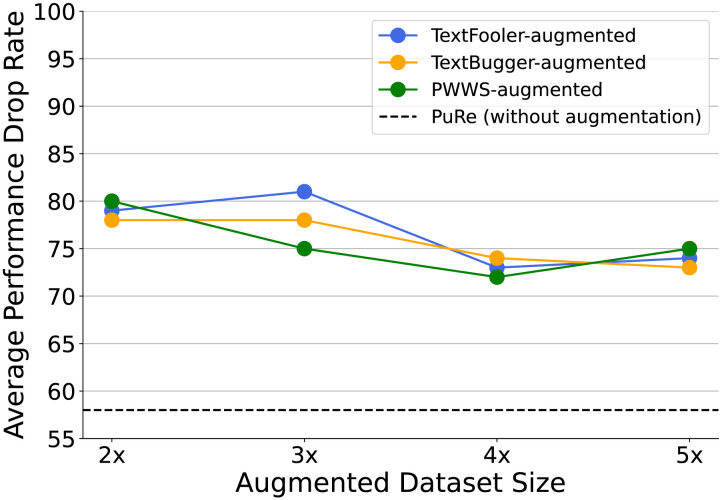
Apdr comparison of the AdvAug training using BERT-base model on the SST2 test set. While AdvAug improves robustness, PuRe achieves a higher Apdr without incurring the computational overhead of generating and incorporating adversarial examples.

As shown in Figure 2, increasing the size of the augmented dataset generally leads to a decrease in Apdr, indicating improved robustness. However, this improvement tends to plateau beyond a certain point, particularly around the 4x and 5x augmentation levels, suggesting diminishing returns from simply scaling up adversarial data. Moreover, despite the increased exposure to adversarial examples, none of the AdvAug configurations are able to match the robustness achieved by PuRe, which attains a substantially lower Apdr of 58.35% without requiring any adversarial examples during training. These findings underscore the efficiency and effectiveness of PuRe, which offers strong adversarial robustness without incurring the computational overhead associated with extensive adversarial data generation and augmentation.

### 5.3 Comparing with Different Models

**Figure 3:**
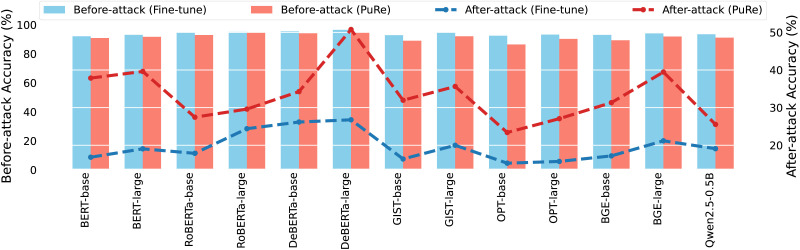
Comparison of before-attack (bar plots) and after-attack (line plots) accuracy on the SST2 test set across various model architectures for both the standard fine-tune baseline and our proposed PuRe approach. The left y-axis shows the models’ performance before adversarial attacks, while the right y-axis shows their performance after attacks. PuRe consistently achieves higher after-attack accuracy while maintaining competitive before-attack performance, demonstrating its enhanced adversarial robustness.

In this section, we compare PuRe with different model architectures. The focus of this setting is not to compare models directly, but to assess the limits and feasibility of PuRe when changing to different model architectures. As seen in Figure 3 and Table 10, PuRe improves adversarial robustness across all architectures, notably providing large performance gain consistently on masked language models (e.g., BERT, DeBERTa). We find that PuRe is less effective for more recent generative-based models like Qwen2.5. We conclude that this is attributed to two factors: (i) larger generative models encode complex feature spaces, with adversarial perturbations spanning multiple principal components, making single-component removal less effective and requiring task- and model-aware mechanisms for optimal balance; and (ii) masked models are trained by predicting a masked token based on its surrounding context, encouraging the model to focus heavily on local context (the nearby words). Any adversarial noise (e.g., small perturbations designed to trick the model) tends to affect only a few specific dimensions of the feature space, making it easier for PuRe to address.

This aligns with findings in Timkey and van Schijndel (2021b), which show that encoder-based models, tend to suffer more from representation degeneration, evidenced by the dominance of a single dimension in their embeddings. For Qwen2.5, we further conducted more detailed experiments (in §5.3.1) to explore the impact of removing additional principal components beyond the top-1 on adversarial robustness.

#### 5.3.1 Removing Additional Principal Components beyond the Top-1

To understand the impact of removing principal components in adversarial robustness, we were motivated by prior findings from Timkey and van Schijndel (2021b), which highlighted a contrast between encoder-based and decoder-based Transformer models in terms of dimensionality dominance. It was found in Timkey and van Schijndel (2021b) that, top-1 dimension dominates the cosine similarity contribution between random sentence pairs for encoder-based models; while on the other hand, top-3 dimensions contribute more equally to GPT-2, which is a finding that we hypothesize could generalize to more decoder-based models. Building on this insight, we conducted experiments to examine whether such patterns hold for latest state-of-the-art decoder models such as Qwen2.5 (Yang et al., 2024; Qwen Team, 2024). Our results confirmed these observations: for encoder-based models (e.g., BERT, RoBERTa, and DeBERTa), removing additional principal components beyond the top-1 caused a marked decline in before-attack accuracy. Conversely, our experiments (see Table 2) with the decoder-based model Qwen2.5 revealed an intriguing behavior: removing more than the top-1 principal component improved after-attack accuracy, albeit with a slight reduction in before-attack accuracy. Removing the top-1 to top-3 components further enhances robustness while maintaining reasonable accuracy (91.16%). However, removing more components (top-4 and top-5) yields minimal robustness gains but a sharp accuracy drop (87.64%). A connection can be drawn from the above findings and Timkey and van Schijndel (2021b): For models that take more dimensions in embeddings to dominate the cosine similarity computation, removing more than one principle components helps bringing an isotropic embedding space, and improved adversarial robustness. However, there exists a trade-off between reaching isotropy and losing too many informative components.

**Table 2:** Ablation study of removing more than the top-1 principal component for Qwen2.5 on SST2 test set. Removing more than just the top-1 principal component can promote a more isotropic embedding space and improve after-attack accuracy; however, excessive removal may degrade performance on clean examples.

**Setting**	**Acc** ↑	**Aua** ↑		
		**TextFooler**	**TextBugger**	**PWWS**
Fine-tune	93.47	5.66	34.71	16.91
PuRe				
Remove top-1 PC	91.43	9.06	34.27	16.91
Remove top-1 to top-3 PCs	91.16	13.18	39.81	21.86
Remove top-1 to top-5 PCs	87.64	13.73	39.32	23.56

### 5.4 Commonsense Reasoning Task

Following prior work (Branco et al., 2021), we adopted only TextFooler to evaluate the adversarial performance under same experimental settings. Table 3 presents the results of various defense methods on commonsense reasoning datasets, using BERT-base as the underlying architecture. We compare PuRe exclusively against regularization-based defense methods, as these approaches do not rely on prior knowledge of the adversary’s synonym generation. Overall, PuRe emerges as a strong adversarial defense method in the context of commonsense reasoning tasks, balancing both before-attack performance and robustness to adversarial perturbations. These findings offer evidence for further exploration of PuRe’s applicability to a wider range of NLP tasks.

**Table 3:** The experiment results on the commonsense reasoning tasks using the BERT-based model.

**Dataset**	**Defense**	**Acc** ↑	**TextFooler**		
			**Aua** ↑	**Asr** ↓	**AvgQ** ↑
SIQA	Fine-tune	61.51	3.68	94.01	41.77
	Flooding-X	61.07	3.64	94.04	41.61
	ALS	**61.76**	4.01	93.51	42.19
	**PuRe (Ours)**	59.57	**11.62**	**80.50**	**51.95**
CSQA	Fine-tune	57.17	3.28	94.26	25.87
	Flooding-X	57.98	3.64	93.72	24.33
	ALS	**58.11**	4.79	91.76	26.76
	**PuRe (Ours)**	55.96	**7.61**	**86.40**	**28.19**

### 5.5 Natural Robustness in PuRe

**Figure 4:**
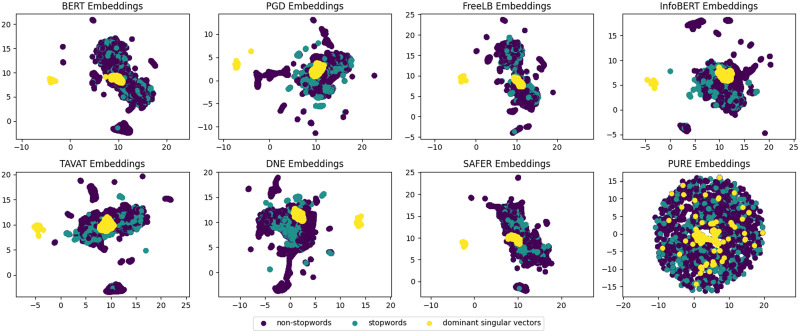
Each token (not each sentence) is projected onto a 2D subspace using UMAP (McInnes et al., 2018). The baselines exhibit anisotropic distributions: Stopword tokens (green) cluster near dominant singular vector directions (yellow), consistent with findings that high-frequency tokens tend to align with top principal components (Arora et al., 2017). This alignment creates predictable directions that adversaries can exploit. In contrast, PuRe disperses both stopwords and dominant components, resulting in a more isotropic distribution that substantially reduces concentrated adversarial attack surfaces.

In this section, we illustrate a key property of PuRe: *natural robustness*. This is termed natural because model’s robustness is achieved without *explicit* adversarial defense methods. First, we discuss the relationship between robustness and isotropy. As depicted in Figure 4, PuRe maps each input sentence to a lower-dimensional space, effectively bringing perturbed and normal sentences into closer proximity in a more uniform distribution. Then, the adversarial examples are somehow treated as normal samples in the embedding space, smoothing the attack. This means that the perturbed parts in adversarial examples will take a weaker effect on the victim models. A parallel can be drawn with the findings

in Arora et al. (2016), which detail that the isotropy has a “purification” effect that mitigates the (rather large) approximation error in the PMI models (Church and Hanks, 1990), and underscores the power of high-dimensional geometry to retain structure through isotropic regularization in embeddings. We further investigate the natural robustness of PuRe by assessing intra-sentence similarity scores (Xiao et al., 2023a), illustrated in Table 4, revealing the isotropic characteristics of PuRe. Specifically, PuRe increases the unadjusted intra-sentence similarity from 0.592 to 0.895, highlighting its effectiveness to induce a more robust and semantically rich sentence-level representation. This isotropy property reduces the likelihood of noise dominating any single direction in the latent space, while preserving meaningful semantic structures. Isotropy in PuRe can be seen as a high-dimensional analog of the Johnson-Lindenstrauss (Johnson, 1984) property, where the post-PuRe contributes equally across dimensions and maintaining the semantic structure of the data.

**Table 4:** Intra-sentence similarity score of last hidden layer, with vanilla fine-tuning and PuRe. For PuRe, we measure both pre-PuRe layer and post-PuRe layer.

**Model**	**Fine-tune**	**PuRe (Pre)**	**PuRe (Post)**
Unadjusted	0.794	0.592	0.895
- Anisotropy Estimates	0.129	0.002	0.008
= Adjusted	0.665	0.590	0.887

### 5.6 PCR and PFSA Modules

In this section, we explore the impact of the PCR and PFSA modules through an ablation study on the SST2 and MRPC datasets, as shown in Table 5. While PCR and PFSA are effective individually, significantly surpassing the baselines (see Table 1), their combined use within PuRe leads to substantial improvements (lower Pdr and higher Aua) in resisting adversarial attacks.

**Table 5:** Ablation study on PCR and PFSA modules.

**Dataset**	**PCR**	**PFSA**	**Acc** ↑	**Aua** ↑		
				**TextFooler**	**TextBugger**	**PWWS**
SST2		✓	**91.98**	16.09	40.20	21.75
	✓		91.71	10.27	35.15	16.75
	✓	✓	90.88	**30.37**	**47.17**	**36.02**
MRPC		✓	83.48	12.35	12.35	15.42
	✓		**84.12**	7.83	10.32	11.65
	✓	✓	82.20	**17.22**	**16.29**	**18.55**

### 5.7 Analysis of rSVD in PCR

One of the most notable findings is the superior performance of rSVD compared to SVD when integrated into PCR, as demonstrated in Table 6. It highlights the Aua and Pdr scores of the BERT-base model using both SVD and rSVD on the SST2 and MRPC datasets, clearly showing the superiority of rSVD.

**Table 6:** Impact of randomization in PCR module on the SST2 and MRPC datasets.

**Dataset**	**Randomized**	**Acc** ↑	**Aua** ↑		
			**TextFooler**	**TextBugger**	**PWWS**
SST2	SVD	90.88	24.49	44.59	30.20
	rSVD	**91.76**	**30.37**	**47.17**	**36.02**
MRPC	SVD	75.77	3.86	6.42	5.13
	rSVD	**82.20**	**17.22**	**16.29**	**18.55**

The inherent stochastic nature of rSVD, which involves the introduction of a Gaussian matrix Ω as described in §3.1.1, introduces a level of randomness that serves as implicit regularization. The randomization in rSVD potentially enhances the model’s robustness. We speculate that this robustness manifests as an increased difficulty for adversaries to craft effective attacks, due to the unpredictable nature of the decomposition’s outcome. This aligns with previous studies (Moon et al., 2023b; Zeng et al., 2023), which have shown the benefits of randomness in improving adversarial defenses.

### 5.8 Run Time Analysis

We compare the computation speed of PuRe with the baselines on the BERT-base model fine-tuned on MRPC because this dataset has the longest average sequence length. All experiments are carried out on a single RTX 4090 GPU. Following prior work (Wang and Lin, 2025), we adjust the number of gradient computation steps for PGD, FreeLB, and InfoBERT to 5, aligning other parameters with the default configurations as specified in their respective original papers. Pre-processing times for DNE and SAFER were excluded to maintain comparability. As shown in Table 7, while fine-tuning serves as a baseline with a run time of 1.0 for both training and inference, PGD and InfoBERT exhibit significantly higher training costs (x3.3 and x3.8, respectively) despite similar inference times. While baselines like Flooding-X and ALS require slightly less runtime than PuRe, their robustness performance is substantially weaker compared to PuRe. Additionally, PuRe offers a more efficient solution, particularly with the rSVD variant. These results (i.e., Table 6 and Table 7) indicate that the randomization in rSVD not only reduces computational costs but also enhances robustness against adversarial attacks, making it a superior choice over standard SVD without any apparent trade-off in accuracy.

**Table 7:** Runtime comparison of PuRe and baseline methods on the MRPC dataset, with Δ Apdr indicating the absolute drop in Apdr relative to the fine-tuning baseline.

**Method**	**Train** ↓	**Inference** ↓	**ΔApdr ↑**
Fine-tune	1.0	1.0	–
PGD (Madry et al., 2019)	× 3.3	× 1.0	10.82
FreeLB (Zhu et al., 2020)	× 2.6	× 1.0	12.07
InfoBERT (Wang et al., 2020a)	× 3.8	× 1.0	4.54
TAVAT (Li and Qiu, 2020)	× 1.6	× 1.0	10.43
SAFER (Ye et al., 2020b)	× 1.1	× 1.0	16.99
DNE (Zhou et al., 2021b)	× 2.5	× 3.0	0.45
Flooding-X (Liu et al., 2022)	× 1.0	× 1.0	3.81
ALS (Yang et al., 2023b)	× 1.1	× 1.0	3.70
AdvFooler (Hoang et al., 2024)	× 1.0	× 1.5	3.71
PuRe (w/ SVD)	× 1.9	× 1.5	13.66
PuRe (w/ rSVD)	× 1.2	× 1.1	17.17

## 6 Conclusion

In this work, we propose a simple yet effective adversarial defense method called PuRe, which has *natural robustness* against adversarial attacks. PuRe is designed as an easily integrable add-on module, based on a straightforward variant of PCA, enabling seamless application to off-the-shelf PLMs with minimal modifications. PuRe was rigorously evaluated across eight diverse language understanding datasets, demonstrating that PuRe not only enhances adversarial defense but also strikes a balance between robustness and generalization. Our evaluation is conducted using the TextAttack framework, focusing on general-purpose attacks relevant to sequence classification and commonsense reasoning tasks. While our evaluation provides strong evidence of effectiveness, future work may consider expanding to newer or more specialized attacks to further validate PuRe’s robustness. Additionally, although PuRe requires only standard fine-tuning (i.e., without the need for adversarial examples or custom regularization), it is not entirely training-free; the PLM still requires fine-tuning with PuRe integrated in order to refine the embedding space for the downstream task. Nevertheless, the simplicity, effectiveness, and compatibility of PuRe highlight its potential as a foundational component for building robust NLP systems.

## Limitations

### Adversarial Attacks.

We assess PuRe’s robustness using TextAttack, a widely used NLP adversarial benchmark that includes methods implemented prior to 2021. While sufficient for general-purpose evaluation, it does not cover newer attacks. As adversarial techniques evolve, future work should incorporate broader evaluations. Additionally, adversarial NLP remains limited in realism: Most perturbations are lexical and less representative of real-world threats compared to imperceptible manipulations in computer vision (Chen et al., 2022).

### Scalability to Larger Models.

Our study is limited to models under 1B parameters due to hardware constraints. Larger models (e.g., 7B+) require significantly more memory and are typically fine-tuned with methods like LoRA (Hu et al., 2022), complicating clean ablations. It is unclear whether PuRe’s gains extend to such models, and we encourage future work to explore its scalability and compatibility with larger architectures.

### Not Training-free.

Although PuRe avoids adversarial training or custom regularizers, it still requires fine-tuning with the module integrated. Thus, it is not training-free and assumes access to model gradients, making it unsuitable for black-box or API-only scenarios.

## Ethics and Broader Impact

The inherent nature of adversarial attacks raises ethical concerns, as malicious users may leverage theoretical adversarial attack literature to develop dangerous tools for the misuse of deployed deep learning systems. It is crucial to emphasize that the present study diverges from proposing novel adversarial attack techniques. Instead, its focus lies in devising a methodology to alleviate the susceptibility of the most vulnerable or adversarial examples within the neural network. Consequently, this specific research endeavor does not give rise to perceived ethical concerns.

## Acknowledgments

We are grateful to the action editor and the anonymous reviewers for their thoughtful feedback and valuable suggestions, which have helped improve the quality and clarity of this work. This work was partially supported by the Economic and Social Research Council (ES/V011278/1) and Engineering and Physical Sciences Research Council (EP/Y009800/1).

## Notes

1 The title “Adversarial Defense without Adversarial Defense” is intended to be paradoxical. It signifies that PuRe enhances adversarial robustness without employing the common strategies of adversarial defense (e.g., generating adversarial examples for training).2 We investigated the impact of removing the top-k principal components, and observed a plummet in before-attack accuracy. Therefore, we set the default to removing only the top-1 principal component. Ablation study can be found in §5.3.1.3 https://github.com/QData/TextAttack.4 https://github.com/PuReDefence/PuRe.5 https://huggingface.co/models.

## A Preliminaries: PCA and SVD

Principal Component Analysis (Abdi and Williams, 2010, PCA) and Singular Value Decomposition (SVD) (Golub and Reinsch, 1971) are cornerstone techniques in the field of machine learning. PCA seeks to transform a set of possibly correlated variables into a smaller number of uncorrelated variables called principal components, with the first principal component accounting for the largest possible variance in the data. This transformation is achieved by identifying the eigenvectors of the data covariance matrix, which correspond to the directions of maximum variance. On the other hand, SVD decomposes a matrix **X** into three distinct matrices

$$U\Sigma V^{⊤}\leftarrow \text{SVD}(X)$$ (3)

where **U** and **V** contain the left and right singular vectors, and **Σ** is a diagonal matrix with singular values. These singular values are crucial as they measure the importance of each corresponding singular vector in capturing the variance of data. The expression SVD(**X**) can also be rewritten as a sum of the outer products of the singular vectors, weighted by the singular values (i.e., linear combination of rank-1 matrices):

$$U\Sigma V^{⊤}=\overset{k}{\underset{i=1}{\sum }}\sigma_{i}u_{i}v_{i}^{⊤}$$ (4)

where **u**_*i*_ and **v**_*i*_ are the columns of **U** and **V** called the left-singular vectors and right-singular vectors, respectively, and *k* is the rank of the matrix **X**. Here, each term $\sigma_{i}u_{i}v_{i}^{⊤}$ represents a rank-1 matrix, and the sum of these rank-1 matrices approximates the original matrix **X**.

Both PCA and SVD are intrinsically related. PCA can be performed through SVD by decomposing the data matrix **X** and then using the singular vectors as the principal components. The elegance of SVD, beyond dimensionality reduction, lies in its ability to provide a mathematically rigorous and computationally efficient method for identifying the underlying structure of data. In §3.1, we propose to use PCA and SVD to enhance the adversarial robustness of NLP models.

## B Datasets

In this paper, we fine-tune PLMs on eight datasets: SST2 (Socher et al., 2013), SUBJ (Pang and Lee, 2004), CR (Hu and Liu, 2004), MR (Pang and Lee, 2005), MRPC (Dolan and Brockett, 2005), SICK (Marelli et al., 2014), SIQA (Sap et al., 2019), and CSQA (Talmor et al., 2019). A detailed description of each dataset is available in Table 8.

**Table 8:** Statistics of datasets. ^*^ Distribution of the examples across classes in validation/test set.

**Dataset**	**Task**	**Classes**	**Train**	**Validation**	**Test**	**Label Distribution^*^**
SST2	Sentiment Analysis	2	6920	872	1821	Approx. Equal
SUBJ	Subjectivity Status	2	8000	–	2000	Approx. Equal
CR	Sentiment Analysis	2	3394	–	376	Approx. Equal
MR	Sentiment Analysis	2	8530	1066	1066	Equal
MRPC	Paraphrase Identification	2	3668	408	1725	Approx. Equal
SICK	Textual Entailment	3	4439	495	4906	Approx. Equal
SIQA	Commonsense Reasoning	3	33410	1954	–	Approx. Equal
CSQA	Commonsense Reasoning	5	9741	1221	–	Approx. Equal

## C Adversarial Defense Baselines

### PGD

(Madry et al., 2018) is a simple method to obtain adversarial perturbations. The process of PGD can be represented as the following min-max problem:

$$\underset{\theta }{\min}E_{(x,y)∼D}\left[\underset{\delta }{\max}L\left(f_{\theta }(x+\delta ),y\right)\right]$$ (5)

where (*x*, *y*) is the data points in the dataset D (*x* is the input sample and y is the corresponding label), *f*_*θ*_(*x*) is the model with parameters *θ*, *δ* is the perturbation added to *x*, and $∥\delta ∥\leq \epsilon$ enforces a constraint on the perturbation budget *ϵ*.

PGD generates the adversarial perturbation *δ* iteratively. For each iteration *t*, update *δ*^(*t*)^ by performing a gradient ascent step *α* to increase the loss.

$$\delta^{\left(t+1\right)}=\delta^{\left(t\right)}+\alpha \cdot \text{sgn}\left(\nabla_{\delta }L\left(f_{\theta }(x+\delta^{\left(t\right)}),y\right)\right)$$ (6)

where $\nabla_{\delta }L\left(f_{\theta }(x+\delta^{\left(t\right)}),y\right)$ is the gradient of the loss with respect to *δ*, and sgn(·) takes the sign of each component in the gradient.

### FreeLB

(Zhu et al., 2020) extends PGD by performing multiple mini-batch updates to craft adversarial examples. That is, it combines the adversarial perturbation with large-batch optimization and reuses gradients across multiple steps, effectively increasing efficiency without requiring separate gradient calculations for each step. It simultaneously accumulates the “free” parameter gradients ∇_*δ*_ℒ in each iteration.

### SAFER

(Ye et al., 2020a) employs randomized smoothing techniques to certify that a model’s prediction remains consistent within a defined radius of perturbation.

$$f_{RS}(X)=\arg\underset{c\in Y}{\max}P_{Z∼\Pi_{X}}\left(f(Z)=c\right)$$ (7)

where *f*_*RS*_ represents the smoothed classifier, **X** is the original sentence, *c* is a class in the label set *Y*, Π_**X**_ denotes the distribution of perturbed sentences around **X**, and *f*(**Z**) is the classifier’s prediction for a perturbed input **Z**. SAFER averages predictions over randomly perturbed versions of the input to certify robustness against adversarial word substitutions.

### InfoBERT

(Wang et al., 2021a) enhances adversarial training by maximising mutual information between clean and adversarial samples, promoting alignment between original and perturbed representations.

$$\max I(Y;T)-n\beta \overset{n}{\underset{i=1}{\sum }}I(X_{i};T_{i})+\alpha \overset{M}{\underset{j=1}{\sum }}I(T_{k_{j}};Z)$$ (8)

where *I*(*Y* ;**T**) is the mutual information between label set *Y* and the learned representation **T**, to retain task-relevant information. $n\beta \sum_{i=1}^{n}I(X_{i};T_{i})$ is the information bottleneck regularizer, which minimizes mutual information between the input **X** and its representation **T**, removing irrelevant or noisy information that could be vulnerable to adversarial attacks. Here, **X**_*i*_ is the word token, and **T**_*i*_ is its local feature representation. $\alpha \sum_{j=1}^{M}I(T_{k_{j}};Z)$ is the anchored feature regularizer, which maximizes mutual information between selected robust local features $T_{k_{j}}$ and the global sentence representation **Z**, aligning stable local features with the global representation.

### TAVAT

(Li and Qiu, 2021) constructs fine- grained virtual adversarial examples by applying perturbations selectively at the token level instead of a rigid normalization ball over the entire sequence. TAVAT can be summarized by the following objective function, which combines instance-level and token-level perturbations:

$$\underset{\theta }{\min}E_{\left(X,y\right)}\left[\underset{∥\delta ∥\leq \epsilon }{\max}\underset{∥\eta_{i}∥\leq n_{i}\cdot \epsilon }{\max}L(f_{\theta }(X+\delta +\eta ),y)\right]$$ (9)

Here, *δ* represents the instance-level perturbation constrained by *ϵ*, and *η*_*i*_ denotes token-level perturbations scaled by *n*_*i*_ for flexibility based on each token’s importance.

### DNE

(Zhou et al., 2021b) employs a neighborhood exploration technique to create virtual sentences by mixing the embedding of the original word in the input sentence with its synonyms. It can be summarized with the following training objective, using virtual examples sampled from the convex hull of a word and its synonyms:

$$\underset{\theta }{\min}E_{(X,y)∼D}\left[\underset{\beta }{\max}L(f_{\theta }(X_{\beta }),y)\right]$$ (10)

where $X_{\beta }=\sum_{x_{j}\in S(x_{i})}\beta_{j}x_{j}$ represents virtual samples in the convex hull spanned by each word *x*_*i*_ and its synonyms *S*(*x*_*i*_), with the weights *β* drawn from a Dirichlet distribution.

### Flooding-X

(Liu et al., 2022) is an efficient and computational-friendly algorithm for improving PLM’s generalization and resistance to adversarial attacks. They theoretically prove that the vanilla Flooding method (Ishida et al., 2020) is able to boost model’s adversarial robustness by leading it into a smooth parameter landscape. If the original learning objective is *J*, then the modified learning objective $\tilde{J}$ with flooding is

$$\tilde{J}(\theta )=|J(\theta )-b|+b,$$ (11)

where *b* > 0 is the flood level and *θ* is the model parameter.

### ALS

(Yang et al., 2023b) applies label smoothing during adversarial training, softening the model’s predictions and reducing sensitivity to adversarial inputs, particularly in out-of-domain contexts. ALS arises from the worst possible smooth label for each input example.

### AdvFooler

(Hoang et al., 2024) randomizes the latent representation of the input at test time to fool the adversary throughout the attack, which typically involves iteratively sampling of discrete perturbations to generate an adversarial sample. It can be mathematically summarized as follows:

$$\;z_{i+1}=h_{l}(z_{i}+\epsilon )$$ (12)

where *z*_*i*_ represents the latent representation of the input at the *i*-th layer, *h*_*l*_ denotes the model’s *l*-th layer function, and ϵ∼N(0,νI) is a Gaussian noise with variance *ν* added to the latent space at each layer to randomize the representation, thereby confusing adversarial attacks.

## D Different Model Architectures

We apply the baselines over a diverse set of model architectures, including encoder-only models such as BERT (Devlin et al., 2018), RoBERTa (Liu et al., 2019b), and DeBERTa (He et al., 2020, 2021a, b); decoder-only models like OPT (Zhang et al., 2022) and Qwen2.5 (Yang et al., 2024; Qwen Team, 2024); and embedding-based models such as BGE (Xiao et al., 2023b) and GIST (Solatorio, 2024) to observe the scalability of PuRe. The baselines are fine-tuned according to their default configurations presented in the respective papers. Table 9 describes the checkpoints available on the HuggingFace Hub.^5^Table 10 is the comparison of different model architectures on the SST2 test set.

**Table 9:** PLMs checkpoints from HuggingFace Hub. **Model**: Lists the names of different PLMs. **Checkpoint**: Specifies the HuggingFace checkpoint name with each model. **Params**: Indicates the number of parameters in each model (in millions, denoted by “M”).

**Model**	**Checkpoint**	**Params**
BERT-base	google-bert/bert-base-uncased	109M
BERT-large	google-bert/bert-large-uncased	335M
RoBERTa-base	FacebookAI/roberta-base	124M
RoBERTa-large	FacebookAI/roberta-large	355M
DeBERTa-base	microsoft/deberta-v3-base	184M
DeBERTa-large	microsoft/deberta-v3-large	435M
BGE-base	BAAI/bge-base-en-v1.5	109M
BGE-large	BAAI/bge-large-en-v1.5	335M
GIST-base	avsolatorio/GIST-Embedding-v0	109M
GIST-large	avsolatorio/GIST-large-Embedding-v0	335M
OPT-base	facebook/opt-125m	125M
OPT-large	facebook/opt-350m	350M
Qwen2.5-0.5B	Qwen/Qwen2.5-0.5B	494M

**Table 10:** Comparison of different model architectures on the SST2 test set.

**Model**	**Params**	**Defense**	**Acc** ↑	**TextFooler**			**TextBugger**			**PWWS**			**Apdr** ↓
				**Aua** ↑	**Asr** ↓	**AvgQ** ↑	**Aua** ↑	**Asr** ↓	**AvgQ** ↑	**Aua** ↑	**Asr** ↓	**AvgQ** ↑	
BERT-base	109M	Fine-tune	92.09	6.32	93.14	87.07	30.37	67.02	41.19	13.78	85.03	127.57	81.73
		**PuRe (Ours)**	90.88	30.37	66.59	134.01	47.17	48.10	58.52	36.02	60.36	139.97	58.35
BERT-large	335M	Fine-tune	93.08	8.84	90.50	98.13	31.63	66.02	43.09	16.69	82.06	130.93	79.53
		**PuRe (Ours)**	91.71	29.87	67.43	143.41	49.75	45.75	56.77	39.21	57.25	144.44	56.81

RoBERTa-base	124M	Fine-tune	94.84	5.88	93.80	93.08	33.06	65.14	43.22	14.61	84.60	132.84	81.18
		**PuRe (Ours)**	92.97	16.14	82.63	110.63	42.78	53.99	49.29	23.45	74.78	135.82	70.47
RoBERTa-large	355M	Fine-tune	95.17	10.43	89.04	109.37	41.85	56.03	46.48	20.92	78.02	138.01	74.36
		**PuRe (Ours)**	94.67	16.80	82.25	115.61	45.19	52.26	52.49	26.85	71.64	138.39	68.72

DeBERTa-base	184M	Fine-tune	95.50	12.96	86.43	108.33	42.06	55.95	44.94	23.50	75.39	137.39	72.59
		**PuRe (Ours)**	94.18	21.31	77.38	122.50	50.36	46.53	51.73	31.08	67.00	139.87	63.64
DeBERTa-large	435M	Fine-tune	96.54	9.88	89.76	104.12	48.22	50.06	48.57	22.19	77.02	139.09	72.28
		**PuRe (Ours)**	94.95	37.78	60.21	156.56	67.60	28.80	60.92	46.90	50.61	151.65	46.54

BGE-base	109M	Fine-tune	93.03	7.69	91.74	89.63	29.71	68.06	40.84	14.11	84.83	129.43	81.54
		**PuRe (Ours)**	89.35	19.11	78.61	119.54	46.07	48.43	47.84	28.78	67.79	135.83	64.94
BGE-large	335M	Fine-tune	94.07	10.27	89.08	98.95	35.86	61.88	42.55	17.41	81.49	133.11	77.48
		**PuRe (Ours)**	91.93	30.81	66.49	136.80	50.03	45.58	53.20	37.51	59.20	143.60	57.09

GIST-base	109M	Fine-tune	92.86	5.82	93.73	87.91	30.64	67.00	40.41	12.58	86.46	129.25	82.40
		**PuRe (Ours)**	89.02	24.22	72.79	127.24	44.26	50.28	48.02	27.40	69.22	134.71	64.10
GIST-large	335M	Fine-tune	94.45	8.40	91.10	96.28	33.77	64.24	42.73	17.79	81.16	133.15	78.83
		**PuRe (Ours)**	92.09	25.48	72.33	129.42	48.11	47.76	52.64	33.28	63.86	139.80	61.32

OPT-base	125M	Fine-tune	92.48	4.56	95.07	83.21	28.50	69.18	40.71	12.63	86.34	127.52	83.53
		**PuRe (Ours)**	86.44	13.67	84.18	104.09	35.42	59.02	47.84	21.09	75.60	129.93	72.93
OPT-large	331M	Fine-tune	93.25	4.72	94.94	83.07	30.42	67.37	40.74	11.97	87.16	128.90	83.16
		**PuRe (Ours)**	90.28	16.31	81.93	112.57	39.87	55.84	48.68	24.99	72.32	135.37	70.03

Qwen2.5-0.5B	494M	Fine-tune	93.47	5.66	93.95	86.62	34.71	62.87	42.39	16.91	81.90	132.36	79.57
		**PuRe (Ours)**	91.43	9.06	90.09	95.18	34.27	62.52	46.39	16.91	81.50	132.21	78.04

### D.1 Out-of-Domain Transferability

In Table 11, PuRe generally shows a competitive Apdr compared to other defense methods when tested in cross-dataset transfer settings, indicating that PuRe adapts well to new domains without sacrificing significant performance, highlighting its transferability.

**Table 11:** Experimental results when the models are trained on the **source** dataset and then transferred to the **target** dataset for testing.

**Dataset**		**Method**	**Acc** ↑	**TextFooler**			**TextBugger**			**PWWS**			**Apdr** ↓
**Source**	**Target**			**Aua** ↑	**Asr** ↓	**AvgQ** ↑	**Aua** ↑	**Asr** ↓	**AvgQ** ↑	**Aua** ↑	**Asr** ↓	**AvgQ** ↑	
CR	SST2	Fine-tune	84.24	3.95	95.31	77.96	23.39	72.23	38.36	8.90	89.44	121.47	85.66
		PGD	82.76	6.70	91.90	89.90	25.81	68.81	41.17	14.22	82.81	129.04	81.17
		FreeLB	83.09	4.89	94.12	86.03	26.58	68.01	40.29	11.70	85.92	126.51	82.68
		InfoBERT	84.73	5.33	93.71	83.79	24.55	71.03	39.34	11.81	86.07	125.50	83.60
		TAVAT	81.71	7.25	91.13	93.81	26.80	67.20	41.16	15.05	81.59	129.37	79.97
		DNE	85.11	14.39	83.09	111.46	27.33	67.86	**51.69**	**28.00**	67.04	104.84	72.66
		SAFER	82.67	8.22	90.05	104.52	31.83	61.60	50.02	23.83	71.25	104.93	74.30
		Flooding-X	84.79	2.47	97.09	74.31	21.09	75.13	37.02	8.57	89.90	122.23	87.37
		ALS	**85.28**	6.21	92.72	86.65	28.01	67.16	40.76	12.19	85.71	123.80	81.86
		AdvFooler	79.91	7.32	90.84	90.44	28.49	64.35	45.54	12.02	84.96	127.77	80.05
		**PuRe (Ours)**	77.65	**21.53**	**72.28**	**123.00**	**39.87**	**48.66**	50.12	27.79	**64.21**	**135.51**	**61.72**
SST2	CR	Fine-tune	**85.37**	2.13	97.51	76.36	28.46	66.67	34.31	9.04	89.41	129.23	84.53
		PGD	80.85	8.78	89.14	87.87	28.99	64.14	35.25	18.09	77.63	134.22	76.97
		FreeLB	84.04	6.65	92.09	85.03	27.93	66.77	35.61	14.63	82.59	133.03	80.48
		InfoBERT	83.78	5.05	93.97	83.83	25.27	69.84	35.05	14.36	82.86	132.41	82.22
		TAVAT	82.18	8.78	89.32	90.23	26.60	67.64	36.67	18.09	77.99	135.77	78.32
		DNE	72.53	13.46	**81.58**	103.14	25.82	64.39	**48.96**	19.23	73.48	110.01	73.15
		SAFER	85.16	10.16	88.06	**105.68**	**35.44**	**58.12**	47.16	**23.08**	**72.73**	109.94	72.97
		Flooding-X	82.45	4.52	94.52	80.03	27.66	66.45	35.39	13.03	84.19	132.11	81.72
		ALS	83.78	4.52	94.60	82.50	29.79	64.44	35.03	9.04	89.21	127.33	82.75
		AdvFooler	80.02	9.93	87.59	99.48	27.71	65.37	45.59	17.39	78.27	130.21	77.08
		**PuRe (Ours)**	82.18	**14.89**	81.88	104.05	33.78	58.90	41.77	22.07	73.14	**136.67**	**71.31**
MR	SST2	Fine-tune	93.68	10.60	88.69	103.33	35.69	61.90	44.93	18.12	80.66	130.63	77.08
		PGD	94.34	17.02	81.96	**130.29**	48.11	49.01	49.54	33.94	64.03	**144.01**	65.00
		FreeLB	94.73	11.53	87.83	114.08	42.01	55.65	47.41	23.39	75.30	138.34	72.93
		InfoBERT	94.73	11.09	88.29	115.44	40.91	56.81	47.05	25.92	72.64	139.05	72.58
		TAVAT	94.34	18.07	80.85	125.73	45.96	51.28	48.71	33.66	64.32	143.25	65.48
		DNE	93.44	10.11	89.18	92.50	23.06	75.27	45.99	21.50	76.99	105.23	80.48
		SAFER	95.22	21.28	77.65	141.70	**50.58**	**46.91**	**58.17**	**44.11**	**53.68**	107.11	**59.41**
		Flooding-X	94.01	5.05	94.63	87.04	28.67	69.51	41.86	13.12	86.04	128.63	83.39
		ALS	95.00	8.79	90.75	105.76	39.98	57.92	44.38	17.08	82.02	132.29	76.90
		AdvFooler	93.56	15.78	83.13	120.01	37.82	59.58	51.34	27.90	70.18	138.33	70.96
		**PuRe (Ours)**	**95.72**	**25.15**	**73.72**	130.26	49.97	47.79	57.10	31.85	66.72	138.69	62.74
SST2	MR	Fine-tune	88.84	6.00	93.24	94.82	27.39	69.17	47.35	13.98	84.27	138.47	82.23
		PGD	89.02	12.20	86.30	123.84	36.68	58.80	52.28	25.98	70.81	149.28	71.97
		FreeLB	88.74	10.51	88.16	119.57	36.12	59.30	53.50	22.98	74.10	148.97	73.85
		InfoBERT	88.84	7.69	91.34	111.08	33.02	62.83	49.83	19.79	77.72	145.65	77.30
		TAVAT	88.84	12.66	85.74	132.17	39.49	55.54	53.71	26.92	69.69	**151.60**	70.32
		DNE	82.87	14.27	82.78	124.83	27.33	66.82	57.06	28.54	65.44	114.46	71.68
		SAFER	**89.82**	13.32	85.17	**136.63**	**44.48**	**50.37**	**61.84**	**38.06**	**57.49**	115.95	**64.34**
		Flooding-X	88.56	3.00	96.61	87.24	24.58	72.25	43.43	12.10	86.33	138.73	85.06
		ALS	89.68	7.13	92.05	103.73	31.80	64.54	47.36	15.85	82.32	140.79	79.64
		AdvFooler	88.44	12.99	85.31	110.30	35.29	60.10	53.39	24.10	72.75	120.68	72.72
		**PuRe (Ours)**	88.65	**18.76**	**78.84**	119.91	37.52	57.67	55.47	24.30	72.59	146.13	69.70

Liu et al. (2022) proposed that “flooding” the loss function by maintaining it near a predefined constant could theoretically improve adversarial robustness by reducing sensitivity to small perturbations. However, our empirical findings indicate that Flooding-X fails to enhance adversarial robustness as anticipated, performing poorly across all robustness metrics in transfer scenarios. This may stem from differences in experimental setups, datasets, or attack methods, or suggest that Flooding’s impact on robustness is more limited than initially claimed. Consistent with Zhu and Rao (2023), we find that Flooding alone does not effectively promote adversarial robustness, despite its potential benefits for reducing overfitting or improving generalization in benign settings.

We observe that SAFER demonstrates good adversarial robustness specifically in transfer settings, likely due to its unique combination of embedding stabilization and randomized smoothing techniques. Embedding stabilization reduces the model’s sensitivity to small perturbations by replacing words with synonyms or perturbing embeddings, which lessens the impact of attacks relying on fine-grained modifications. Furthermore, randomized smoothing adds noise to the embedding space, making the model’s outputs less predictable and increasing the query cost for attackers. This approach allows SAFER to effectively generalize to new datasets in transfer scenarios, as its robust representations extend well beyond the original training data, offering enhanced protection without requiring adversarial training tailored to each attack type.

The performance of PuRe is compared against various baseline methods across multiple adversarial robustness metrics. Although PuRe does not achieve the highest performance across all individual metrics, it demonstrates competitive results, achieving either the best or second-best Apdr outcomes. This suggests that PuRe provides a robust balance in terms of adversarial resilience, making it an effective approach for general-purpose robustness (Table 1), even when evaluated on transfer datasets (Table 11).

## E PFSA Hyperparameter Analysis

We performed a hyperparameter sensitivity analysis for the PFSA module to identify optimal values for the sampling parameter *r* and scaling factor *α*, as detailed in Table 12. *r* = 8 and *α* = 1.5 were adopted as the optimal hyperparameters for the PFSA module on the SST2 dataset.

**Table 12:** Impact of *r* (a sampling parameter indicating the number of Gaussian random vectors mentioned in §3.1.1) and *α* (a scaling factor used to enhance feature expression mentioned in Zhai et al. [2023]) with BERT-based model on the SST2 dataset.

**Hyper-parameter**		**Acc** ↑	**TextFooler**			**TextBugger**			**PWWS**		
**Key**	**Value**		**Aua** ↑	**Asr** ↓	**AvgQ** ↑	**Aua** ↑	**Asr** ↓	**AvgQ** ↑	**Aua** ↑	**Asr** ↓	**AvgQ** ↑
*r*	8	90.88	**30.37**	**66.59**	**134.01**	47.17	48.10	**58.52**	**36.02**	**60.36**	**139.97**
*r*	16	92.15	27.84	69.79	132.42	**48.54**	**47.32**	55.74	31.96	65.32	137.25
*r*	32	91.48	25.70	71.89	127.82	45.80	49.91	57.49	31.91	65.11	137.30
*α*	0.5	91.43	17.46	80.89	105.92	39.21	57.09	50.57	23.94	73.80	132.40
*α*	1.0	91.32	23.28	74.50	121.36	43.82	52.01	53.86	28.56	68.73	135.16
*α*	1.5	90.88	**30.37**	**66.59**	**134.01**	**47.17**	**48.10**	**58.52**	**36.02**	**60.36**	**139.97**
*α*	2.0	90.50	20.48	77.37	113.14	38.77	57.16	49.73	26.74	70.45	133.81
*α*	2.5	90.39	18.67	79.34	114.26	41.85	53.71	51.17	26.25	70.96	134.17

## F Further Discussion on the Limitations and Scope of PuRe

While the main body of the paper outlines the primary limitations, this section provides a further discussion on the boundary conditions and potential challenges of PuRe. These points represent important directions for future investigation.

### F.1 The Nature of the Top Principal Component

PuRe’s core hypothesis is that the top principal component (PC) of instance-level embeddings primarily capture common, non-discriminative information (e.g., syntactic patterns, high-frequency word effects) that adversaries exploit. While our results strongly support this for the tasks and models tested, this assumption may not hold universally.

- **Task-Dependent Information:** For certain complex tasks, the top PC might encode discriminative semantic information. For example, in a legal text classification task, a dominant component might represent a key legal concept. In such cases, removing it could harm clean accuracy more significantly than observed in our experiments.
- **Characterizing PCs:** Future work could focus on methods to automatically characterize the information contained within the top PCs for a given task before deciding to remove it. This could lead to an adaptive version of PuRe that only applies the removal when the top PCs is identified as “noise” rather than “signal.”

### F.2 Task- and Model-Specificity of Optimal PCR

Our default implementation removes the top-1 PC, which proved highly effective for encoder-based models. However, as shown in our ablation study with Qwen2.5 (§5.3.1), decoder-based models may benefit from removing additional components (e.g., top-3). This highlights that the optimal number of components to remove is likely not a universal constant but depends on:

- **Model Architecture:** Decoder-only and encoder-decoder architectures may distribute information across their embedding dimensions differently than encoder-only models, leading to different anisotropy patterns.
- **Task Complexity:** Simpler tasks like sentiment analysis might have a single, highly dominant “noise” component, whereas more complex reasoning tasks might have vulnerabilities distributed across several top components.

A “one-size-fits-all” approach may therefore be suboptimal. A more advanced implementation of PuRe could involve a mechanism to dynamically determine the optimal number of PCs to remove based on the model and task.

### F.3 Potential Vulnerability to Adaptive Adversaries

Our evaluation uses established, general-purpose attackers. A more sophisticated, adaptive adversary who is aware of the PuRe defense mechanism could potentially circumvent it. Such an adversary could formulate a new attack by solving an optimization problem with an added constraint: the resulting perturbation must lie in a subspace orthogonal to the top PCs that PuRe removes.

While this would be a significantly harder attack to craft—especially with the added randomness from rSVD—it is theoretically possible. Validating PuRe against such adaptive, white-box attacks would be a critical next step to fully assess its robustness in worst-case scenarios.

### F.4 Applicability to Generative and Open-Ended Tasks

This work focuses on discriminative NLP tasks (e.g., text classification, natural language inference, commonsense reasoning). The applicability of PuRe to generative tasks like text summarization, machine translation, or dialogue systems remains an open question.

- **Information vs. Fluency Trade-off:** Generative models rely on the richness of the representation space to produce diverse, fluent, and coherent text. The information removal inherent in PuRe, while beneficial for robustness in classification, might inadvertently “flatten” the representation space, leading to more generic or stylistically bland text generation. The very components that PuRe removes might be responsible for encoding subtle details crucial for high-quality generation.
- **Evaluation Challenges:** Evaluating the impact on generation quality is also more complex than measuring accuracy and requires a different set of metrics in an adversarial setting.

Future research should explore whether PuRe can be adapted for generative tasks, perhaps by applying it more selectively or with a lower intensity, to strike a balance between robustness and generation quality. Further, it will be interesting to theoretically understand how PuRe provides the implicit robustness to text adversarial attacks and mitigates over-confident predictions on the adversarially attacked examples.
